# Cotidiano e desafios da enfermagem em unidades hospitalares COVID-19: perspectiva dos profissionais*

**DOI:** 10.15649/cuidarte.2600

**Published:** 2023-09-03

**Authors:** Alexa Pupiara Flores Coelho Centenaro, Andressa de Andrade, Clarice Alves Bonow, Marta Cocco da Costa, Rosangela Marion da Silva, Jesica Johanna Rincón Sepúlveda

**Affiliations:** 1 . Universidade Federal de Santa Maria, campus Palmeira das Missóes/RS. Brasil. E-mail: alexa.coelho@ufsm.br Universidade Federal de Santa Maria Universidade Federal de Santa Maria Palmeira das Missóes RS Brazil alexa.coelho@ufsm.br; 2 . Universidade Federal de Santa Maria, campus Palmeira das Missóes/RS. Brasil. E-mail: andressa@ufsm.br Universidade Federal de Santa Maria Universidade Federal de Santa Maria Palmeira das Missóes RS Brazil andressa@ufsm.br; 3 . Universidade Federal de Pelotas/RS. Brasil. E-mail: claricebonow@gmail.com Universidade Federal de Pelotas Universidade Federal de Pelotas RS Brazil : claricebonow@gmail.com; 4 . Universidade Federal de Santa Maria, campus Palmeira das Missóes/RS. Brasil. E-mail: marta.c.c@ufsm.br Universidade Federal de Santa Maria Universidade Federal de Santa Maria Palmeira das Missóes RS Brazil marta.c.c@ufsm.br; 5 . Universidade Federal de Santa Maria/RS. Brasil. E-mail: cucasma@terra.com.br Universidade Federal de Santa Maria Universidade Federal de Santa Maria RS Brazil cucasma@terra.com.br; 6 . Maestría en Ciencias, Universidade Federal de Pelotas/RS, Brasil. Universidad de Pamplona, Colombia. E-mail: jessik2015@hotmail.com Universidad de Pamplona Universidad de Pamplona Colombia jessik2015@hotmail.com

**Keywords:** Covid-19, Enfermagem, Pandemias, Profissionais de Enfermagem, Saúde do Trabalhador, Covid-19, Nursing, Pandemics, Nurse Practitioners, Occupational Health, Covid-19, Enfermería, Pandemias, Enfermeras Practicantes, Salud Laboral

## Abstract

**Introdujo:**

O trabalho de enfermagem em unidades hospitalares COVID-19 é complexo, desafiador e repleto de elementos cuja compreensáo é importante para o campo da Gestáo e Saúde no Trabalho.

**Objetivo:**

compreender o cotidiano e os desafios de trabalhadores de enfermagem na linha de frente do enfrentamento a pandemia em unidades hospitalares COVID-19.

**Materiais e Métodos:**

estudo qualitativo desenvolvido com 35 trabalhadores de enfermagem em unidades COVID-19 de sete hospitais do Sul do Brasil, por meio de entrevistas semiestruturadas. O software NVivo auxiliou no tratamento dos dados a partir da análise temática de conteúdo.

**Resultados:**

da análise dos dados emergiram duas categorias analíticas: Desafios do cotidiano de enfermagem nas unidades COVID-19: complexidade e demandas da assistencia; e Desafios de ser um trabalhador de enfermagem da linha de frente: desdobramentos no bem-estar profissional e na vida pessoal.

**Discussao:**

pode-se considerar que a complexidade e intensificado do trabalho na linha de frente causou impactos que podem conduzir os trabalhadores de enfermagem ao adoecimento. Sáo importantes a0es de promodo a visibilidade profissional junto a sociedade para desmistificar a imagem romantizada sobre a profissáo e problematizar a importancia da enfermagem no enfrentamento da pandemia e os impactos sofridos por estes trabalhadores.

**Conclusao:**

trabalhadores de enfermagem de unidades COVID-19 vivenciam desafios complexos, com repercussóes em sua experiencia laboral e em sua vida.

## Abstract (en)

**Introduction:**

The nursing work in COVID-19 hospital units is complex, challenging, and full of elements whose understanding is important for the field of Management and Health at Work.

**Objective:**

to understand the daily life and challenges of nursing workers in the front line of confronting the pandemic in COVID-19 hospital units.

**Materials and Methods:**

qualitative study developed with 35 nursing workers in COVID-19 units from seven hospitals in southern Brazil, through semi-structured interviews. The NVivo software helped in the data treatment from the thematic content analysis.

**Results:**

two analytical categories emerged from data analysis: Challenges of nursing daily life in COVID-19 units: complexity and demands of assistance; and Challenges of being a frontline nursing worker: unfolding in professional well-being and personal life.

**Discussion:**

it can be considered that the complexity and intensification of frontline work caused impacts that can lead nursing workers to illness. Actions to promote professional visibility in society are important, to demystify the romanticized image about the profession, and to problematize the importance of nursing in facing the pandemic and the impacts suffered by these workers.

**Conclusion:**

nursing workers in COVID-19 units experience complex challenges, with repercussions on their work experience and their lives.

## Resumen

**Introducción:**

El trabajo de enfermería en las unidades hospitalarias COVID-19 es complejo, desafiante y lleno de elementos cuya comprensión es importante para el campo de la Gestión y Salud Ocupacional.

**Objetivo:**

comprender el cotidiano y los desafíos de los trabajadores de enfermería en la primera línea de enfrentamiento a la pandemia en las unidades hospitalarias de la COVID-19.

**Materiales y Métodos:**

estudio cualitativo desarrollado con 35 trabajadores de enfermería en unidades de COVID-19 de siete hospitales del sur de Brasil, a través de entrevistas semiestructuradas. El software NVivo ayudó en el procesamiento de datos basado en el análisis de contenido temático.

**Resultados:**

del análisis de los datos surgieron dos categorías analíticas: Desafíos cotidianos de la enfermería en las unidades de COVID-19: complejidad y demandas asistenciales; y Desafíos de ser un trabajador de enfermería de primera línea: desarrollos en el bienestar profesional y personal.

**Discusión:**

se puede considerar que la complejidad e intensificación del trabajo en primera línea provocó impactos que podrían llevar a los trabajadores de enfermería a la enfermedad. Las acciones de promoción de la visibilidad profesional en la sociedad son importantes para desmitificar la imagen romantizada de la profesión y discutir la importancia de la enfermería en el enfrentamiento de la pandemia y los impactos sufridos por estos trabajadores.

**Conclusión:**

los trabajadores de enfermería de las unidades de COVID-19 viven desafíos complejos, con repercusiones en su experiencia laboral y en su vida.

## Introdujo

No Brasil, as equipes de enfermagem que atuam nos hospitais sao formadas, majoritariamente, por enfermeiros (profissionais de nível superior) e técnicos de enfermagem (profissionais de nível médio)^1^. Eles desempenham o papel de ofertar cuidados complexos, de alta qualidade e ininterruptos^2^. Por essa razao, em 2019 a Organizado Mundial de Saúde, em conjunto com o Conselho Internacional de Enfermeiros (CNE), langou a campanha "Nursing Now". A iniciativa buscou promover no mundo discussóes em torno da importancia da enfermagem para os desafios em saúde do século XXI, com o objetivo de fomentar agóes direcionadas a potencializagao da educagao, condigóes de trabalho, Saúde do Trabalhador e disseminagao de práticas exitosas da profissao^3^.

Esta campanha coincidiu com o advento da COVID-19, doenga causada pelo vírus Sars-CoV-2. A doenga, que teve surgimento na China, é transmitida por inalagao ou contato com gotículas contaminadas e é caracterizada pela síndrome respiratória aguda grave. A COVID-19 ganhou proporgóes de grande crise de saúde pública, configurando-se como pandemia^4^. No Brasil, medidas foram implementadas para o controle da doenga, incluindo a organizagao de infraestrutura hospitalar capacitada^5^, ou seja, servigos que fazem parte do que se denominou linha de frente do enfrentamento a pandemia.

Trabalhadores de enfermagem compóem essa linha de frente. Além de atuarem nos cuidados aos doentes graves da COVID-19, promovem prevengao, educagao em saúde e combatem a disseminagao de notícias falsas. Porém, ao mesmo tempo, enfrentam um conjunto de riscos e danos relacionados a este trabalho^6^^-^^7^.

O advento da pandemia causada pela COVID-19 colocou a enfermagem em evidencia para a sociedade, devido seu papel no enfrentamento da crise sanitária, o que, de se certa forma, contribuiu para a visibilidade da profissao. No entanto, é importante analisar criticamente o movimento midiático que atribuiu aos trabalhadores a figura de "anjos" ou "heróis da linha de frente"^8^, pois ao mesmo tempo vivenciaram um aumento de problemas físicos e psicossociais como consequencia de sua atuagao nos servigos de saúde^6^.

A literatura tem abordado aspectos relacionados aos impactos deste trabalho na saúde mental e na vida dos trabalhadores de enfermagem; no entanto, sao importantes estudos que resgatem os aspectos humanos dos trabalhadores que atuaram na linha de frente^9^, no sentido de fornecer registros da realidade vivenciada por eles em diferentes contextos. Estes dados serao importantes para o campo da Gestao e Saúde no Trabalho, visando a promogao a saúde mental e reorganizagao do processo de trabalho em saúde e enfermagem, sobretudo no período pós-pandemico.

Sendo assim, o presente estudo teve como objetivo compreender o cotidiano e os desafios de trabalhadores de enfermagem na linha de frente do enfrentamento a pandemia em unidades hospitalares COVID-19.

## Materiais e Método

Trata-se de um estudo qualitativo multicentrico, realizado em sete instituigóes hospitalares localizadas no estado do Rio Grande do Sul, Brasil. Eram quatro instituigóes de grande porte e tres de médio porte. Cinco eram de caráter filantrópico; duas se caracterizavam como hospitais públicos vinculados a instituigóes de ensino federais. Os cenários de estudo abrangeram unidades responsáveis pelo atendimento de casos suspeitos ou confirmados de COVID-19, totalizando: uma unidade de triagem respiratória; cinco setores de urgencia e emergencia; quatro unidades de internado clínica; e quatro unidades de terapia intensiva (UTI's).

Foram incluídos trabalhadores de enfermagem lotados nestas unidades. Foram excluídos trabalhadores em férias ou apresentando qualquer tipo de afastamento funcional do trabalho. As gerencias dos hospitais encaminharam a equipe de pesquisa listas de profissionais que respondiam aos critérios de elegibilidade, com nomes, e-mails e/ ou números de telefone. Um total de 470 trabalhadores compuseram estas listas. Para a etapa de campo, optou-se pela realizado de sorteios aleatórios simples que contemplassem cinco trabalhadores de cada instituigáo, independentemente de suas unidades de lotagáo. Desta forma, participaram do estudo um total de 35 trabalhadores de enfermagem.

Doze trabalhadores náo responderam aos pesquisadores, mesmo quando contatados por tres vezes. Cinco profissionais se recusaram a participar do estudo. Nesses casos, ocorriam novos sorteios até que se viabilizasse o alcance da amostra adequada.

Os dados foram produzidos entre setembro de 2020 e julho de 2021. Utilizou-se a técnica da entrevista semiestruturada, previamente agendadas por e-mail. Os encontros foram organizados de forma presencial em cinco hospitais, com atendimento de todas as medidas de precaugáo contra a disseminagáo da COVID-19.

Previamente ao início da entrevista, apresentou-se o estudo e solicitou-se a assinatura do Termo de Consentimento Livre e Esclarecido (TCLE). Em seguida, conduziu-se o encontro registrando-se informagóes inerentes a caracterizado dos participantes, entre estas, genero, idade, cor/raga, categoria profissional, unidade/setor em que se encontrava lotado. Procedia-se entáo com a entrevista em profundidade seguindo-se um roteiro constituído pelos seguintes tópicos: percepgáo do participante sobre seu trabalho na unidade COVID-19; rotina e cotidianos na assistencia aos pacientes; desafios e dificuldades enfrentados neste cotidiano. As entrevistas foram audiogravadas com a concordancia de todos os participantes.

Duas instituigóes hospitalares solicitaram que as entrevistas fossem conduzidas de forma online. Nestes casos, os entrevistados receberam o TCLE de forma online pelo Google Forms, assinalando sua concordancia. Estas entrevistas foram conduzidas via Google Meet, plataforma gratuita que permite a interlocugáo em tempo real, com compartilhamento de áudio e imagem. Os encontros online foram conduzidos da mesma forma que os realizados presencialmente. Foram audiogravados, ativando-se este recurso na própria plataforma Google Meet.

A equipe de pesquisa foi composta por enfermeiros docentes e estudantes de mestrado com experiencia em pesquisa qualitativa. O tempo médio de duragáo das entrevistas foi de 22,5 minutos.

A transcrigáo das entrevistas compos o corpus do estudo. Para o tratamento dos dados, utilizou-se análise temática de conteúdo, constituída por tres etapas: pré-análise; exploragáo do material; tratamento dos dados obtidos e interpretagáo^10^.

Na pré-análise, foi realizada a imersáo por meio de leitura flutuante, a qual possibilitou a apreensáo do conteúdo e selegáo do material apropriado para análise^10^. Na exploragáo do material, procedeu-se a decomposigáo e codificagáo do material em Unidades de Registro (UR) que sáo termos que traduzem o conteúdo de cada fragmento de fala conforme temas compatíveis com o objetivo de estudo^10^. Contou- se com o auxílio do software New NVivo Academic, procedendo o agrupamento de categorias analíticas.

Por fim, no tratamento dos dados obtidos e interpretado, os pesquisadores avaliaram os resultados e realizaram interpretares e inferencias que conduziram as principais conclusóes^10^.

O banco de dados do estudo multicentrico foi armazenado no Mendeley Data^11^. Nos resultados deste estudo, os participantes estáo identificados pela letra T, que antecede a palavra "trabalhador", seguida por um número cardinal que representa a ordem em que o depoente foi entrevistado. Há também a identificado da unidade de lotagáo: Urgencia/Emergencia ou Unidades de Triagem Respiratória COVID-19 (Emerg. COVID-19); Unidades de Internado COVID-19 (Intern. COVID-19); Unidade de Terapia Intensiva COVID-19 (UTI COVID-19); e mais de um setor COVID-19 (Setores COVID-19).

Foram observados os preceitos éticos direcionados a pesquisa que envolve seres humanos, detalhados nas Resolugóes n° 466/2012 e n° 510/2016, do Conselho Nacional de Saúde. O projeto obteve aprovagáo de um comite de ética em pesquisa local, por meio do parecer n° 4.549.077.

## Resultados

Ao todo, 35 trabalhadores de enfermagem de unidades COVID-19 participaram da pesquisa. Dentre eles, 80% (n=28) eram mulheres. A média de idade foi de 38 (±8,9) anos. No que tange a cor/raga, 77% (n=27) eram brancos e 23% (n=8), pretos ou pardos. No que diz respeito a categoria profissional, 71,4% (n=25) eram técnicos em enfermagem e 28,6% (n=10), enfermeiros.

No que diz respeito as unidades de lotagáo, 42,8% (n=15) estavam em unidades de Urgencia/ Emergencia ou Unidades de Triagem Respiratória COVID-19; 31,4% (n=11), em Unidades de Internagáo COVID-19; 22,8% (n=8), em UTI's COVID-19; e 2,8% (n=1), em mais de um setor COVID-19.

Os resultados foram organizados em duas categorias analíticas, apresentadas a seguir.

### Desafios do cotidiano de enfermagem nas unidades COVID-19: complexidade e demandas da assistencia

O cotidiano da enfermagem nas unidades COVID-19 era marcado por grandes demandas, superlotagáo dos setores e precarizagáo das condigóes de trabalho. Os desafios dessa conjuntura se potencializaram nos períodos de surto da pandemia:

*[...] é a demanda, é UTI cheia, a unidade cheia, pacientes de UTI na unidade porque nao tem leito. Sensagao de impotencia. Nao tem mais material, nao tem leito em lugar nenhum. [...] (T10- Setores COVID-19)*

*[...] no mes de julho, o mais conturbado, eu trabalhava 24 horas. Tinha profissionais contaminados. (T14- Emerg. COVID-19)*

Na voz dos trabalhadores, a rotina nessas unidades era marcada por complexidade, imprevisibilidade e tensáo. Os casos clínicos eram diversos e singulares:

*[...] nenhum dia é igual ao outro [...] teve plantoes em que durante a madrugada houve quatro internares, cinco internares. Teve óbito. Teve aquele paciente que chegou muito bem e que em questao de horas piorou muito. Teve aquele paciente com 90 anos, com 96 anos, teve jovem com 18, com 15 anos [...] cada vida que passou lá foi de uma forma diferente [...] muitas pessoas chegam revoltadas, nao conseguindo acreditar que foram contaminadas pelo virus, chegam pessoas desorientadas... Sao muitas peculiaridades [...] (T33- Intern. COVID-19)*

*Os* trabalhadores destacaram a necessidade de vigilancia constante que os doentes por COVID-19 demandavam. No curso da pandemia as equipes adquiririam maior domínio técnico da doenga, porém com muitas incertezas quanto ao prognóstico:

*[...] na última noite havia cinco pacientes no tubo e uma paciente saturando 38%. Tem que estar sempre ligado. Numa noite o doutor intubou um, saiu daquele e já parou para intubar outro. Temos que ter aquela visao, de olhar para o monitor e saber o que o paciente precisa [...] (T8-UTI COVID-19)*

*[...] hoje já trabalhamos com mais certeza, com mais seguranza. Por mais que continuemos sem saber ao certo o que pode acontecer [...], hoje já temos uma base. [...] (T33- Intern. COVID-19)*

Somavam-se questóes para além da COVID-19 em si, como vulnerabilidades económicas e sociais:

*[...] [paciente] cheio de “bicho de pé”. Horrível. [...] quando eu cortei a unha dele cheirava podre. Ele chegou retraído, acho que ele tinha vergonha [...] a gente pega muito esse tipo de situando. Tem pacientes que sao muito pobres [...] (T8-UTI COVID-19)*

O cotidiano de convívio com os doentes por COVID-19 também esteve presente nas narrativas dos participantes. Foi destacada a construyo dos vínculos ao longo do processo de hospitalizado, que se por um lado proporcionava gratificado e reconhecimento, por outro lado mobilizava emocionalmente os profissionais ante o sofrimento e morte dos pacientes:

*[...] se o paciente estiver bem, falando, tem a chamada de vídeo que fazemos [...] daí a gente se apega [...] porque tem uns que já faz vinte e poucos dias que estao conosco e toda a tarde fazemos chamada de vídeo [...] as vezes as pessoas mandam doce, mandam coisas para nós [...] (T6-UTI COVID-19) [...] é muito difícil porque eu nao quero perder a pessoa. 90% dos [pacientes] chegam falando como eu estou falando contigo e infelizmente eles progridem para um final, um sofrimento. Outras pessoas eu vejo que ficarao sequeladas. Entao tem muita história. Todo mundo chega com uma história [...] (T8-UTI COVID-19)*

No entanto, nem sempre esse vínculo entre profissional e paciente/família se estabelecia, sobretudo nas unidades abertas (Urgencias e Emergencias e Unidades de Triagem Respiratória), em que o cotidiano era marcado pela insatisfagáo dos profissionais em relado ao mau uso que parte da populado fazia destas estruturas:

*[...] muitos pacientes precisam estar aqui, mas está rolando muita brincadeira. As vezes querem só pegar um atestado, numa segunda-feira. Aqui é bem complicado. [...] Parece as vezes que o meu trabalho está sendo insignificante [...] (T14- Emerg. COVID-19)*

Por fim, foi possível perceber que os desafios do enfrentamento a pandemia extrapolavam a realidade do setor de trabalho. Incluíam os dilemas do sistema de saúde, algumas vezes, ineficiente no fluxo de atendimento aos casos da COVID-19:

*[...] alguns pacientes chegam mal manejados, entáo já nao tem muito o que fazer. Chegam tarde. As vezes porque deveriam ter chegado antes. Daínáo tem como fazer milagre. E isso vai me frustrando, vai me cansando. [...] Eu sofro junto [...] (T9-UTI COVID-19)*

### Desafios de ser um trabalhador de enfermagem da linha de frente: desdobramentos no bem- estar profissional e na vida pessoal

*A* estruturagáo das unidades COVID-19 exigiu mudanzas nas contratares e realocagáo de profissionais. Isso desagregou as equipes, impactando nas relagóes e nos vínculos de amizade e afetividade:

*[...] sáo muitas pessoas do contrato, eu náo tenho esse vínculo com eles. Eu sinto falta de ter equipe novamente. De olhar para o lado e saber que o plantáo é muito ruim, que aquela situado é muito ruim, mas eu conhego o meu colega, eu posso contar com ele. O pessoal do contrato náo é assim,*

*[...] eles estáo ali para ganhar um extra. Sáo bem importantes, mas pela questáo do vínculo, gera um pouco de distanciamento. Eu sinto falta de amizade dentro do trabalho [...] (T34-UTI COVID-19)*

Os participantes também relatavam como desafio as medidas de controle contra a disseminagáo da COVID-19. Essas medidas envolviam uma rotina exaustiva que interferia nos horários de lanche, repouso e das próprias necessidades fisiológicas, além de, algumas vezes, culminar em sobrecarga e tensáo:

*[...] tinha os 15 minutos de intervalo, agora a gente náo tem mais porque a contaminagáo está acontecendo muito. O nosso hospital é bem rígido. Na hora do lanche, o máximo que estáo permitindo é dois [profissionais na sala de lanche]. A contaminagáo é muito fácil na hora de tirar os EPI's. A minha máe é grupo de risco, daí eu evito ir e sair [...] é assim a linha de frente, puxado [...] (T6-UTI COVID-19)*

*[...] tem que ficar seis horas lá dentro [unidade COVID-19]. Náo pode ir no banheiro, náo pode comer, isso é bem complicado. A gente já se preparava antes, quando sabia que tinha que entrar no COVID-19: náo tomar muita água de manhá, náo tomar muito chimarráo, almogar bem. Porque a gente náo pode sair de lá nem para comer, nem para ir ao banheiro. [...] se a gente náo estava bem, a gente trocava com um colega. Porque tem que entrar bem ali. Bem preparado [...] (T15- Intern. COVID-19)*

A rotina exaustiva nas unidades COVID-19 resultava em sensagáo de cansago, exaustáo e vontade de abandonar a linha de frente:

*[...] muitas vezes [o trabalho] é hostil. Porque sáo muitos pacientes. Eu trabalho somente lá [unidade COVID-19], mas tem colegas que trabalham em duas unidades, dois hospitais. Entáo é cansativo. É exaustivo. A gente fica todo paramentado e isso cansa. Muitas vezes tem colegas que tem vontade de desistir, porque estáo cansados, porque é uma rotina cansativa [...] (T35- Intern. COVID-19)*

Fora do espago do trabalho, havia situagóes de preconceito por parte da comunidade, familiares e colegas de outros empregos:

*[...] a gente ia no supermercado, a gente ouvia até algumas piadas. Ficavam com receio de nós [...] quando a gente ia em qualquer lugar, por exemplo, na casa de um parente, náo descia do carro*

*[...] eu trabalho na atengáo básica. Chegou um momento em que eu fui na Secretaria de Saúde e conversei com a coordenadora para ela me liberar por um determinado tempo porque eu notei um receio muito grande dos meus colegas, que temiam ficarem próximos de mim [...] eu passava em casa, tomava banho, trocava de roupa e ia para a unidade [...] (T25- Intern. COVID-19)*

Ainda no que diz respeito a vida fora do trabalho, os profissionais de enfermagem reconheceram também as implicates negativas da sua atuagáo na linha de frente em suas dinámicas familiares e em seus projetos de vida:

*[...] eu tenho uma filha pequena, fiquei uns dias afastada dela. Ela ficou na máe. A gente tem medo por ela ser pequena. Eu tinha muito medo. [...] isso pesou muito. O medo de pegar e passar para a familia. Fiquei uns dois meses, quase tres sem ir para casa, sem ver eles. [...] (T15- Intern. COVID-19)*

*[...] estou fazendo doutorado. [...] eu tento chegar em casa e escrever, mas náo rende da mesma forma quando estou com uma carga de trabalho táo grande. [...] eu tinha um plano, que era defender o doutorado até o final desse ano, mas náo vai acontecer. É frustrante [...] (T21- Emerg. COVID-19)*

Por fim, alguns participantes refletiram e reconheceram suas próprias vulnerabilidades, a despeito do imaginário de heroísmo que se construiu socialmente em torno da figura do trabalhador de saúde da linha de frente:

*[...] todos da saúde brincam “sou herói”, “vamos vencer isso e vencer aquilo”. A gente náo ve que atrás da gente tem uma familia, que as vezes está prestes a se quebrar. E a gente ve que náo é herói de nada. A gente é só um médico, uma enfermeira, um técnico... Lutando contra um ser invisível que a gente nem sabe o que é [...] (T7- Intern. COVID-19)*

## Discussao

A primeira categoria analítica mostrou que o cotidiano de enfermagem nas unidades COVID-19 era difícil, desafiador e se agravava nos períodos de surto pandémico, em que os trabalhadores referiram vivenciar piora de suas condigóes de trabalho, devido aos déficits de insumos para a assisténcia, superlotagáo e aumento da jornada de trabalho. O cotidiano da enfermagem no enfrentamento da COVID-19 é marcado por condigóes laborais precarizadas devido a caréncias materiais e de recursos humanos, sobrecarga, descontinuidades no processo de trabalho e fragmentagáo da gestáo do cuidado^12^^-^^14^.

Foi destacada a complexidade, imprevisibilidade e tensáo que caracterizaram as unidades COVID-19 e o perfil clínico destes pacientes. Sabe-se que os casos de infecgáo pelo Sars-CoV-2 sáo inespecíficos e diversificados, variando entre casos leves, moderados e graves, com complicagóes pulmonares e extrapulmonares, acometendo diferentes perfis populacionais e apresentando diferentes possibilidades de desfechos clínicos^15^.

Nas unidades COVID-19, a enfermagem assume um conjunto de agóes complexas. Citam-se a gestáo do cuidado e do processo de trabalho nestes setores, reconhecimento de sinais e sintomas, vigiláncia e assisténcia respiratória, detecgáo precoce de sinais de agravamento clínico, manejo clínico, nutrigáo, hidratagáo, promogáo do sono e repouso, preparo da alta. A isso, somam-se os cuidados paliativos, procedimentos pós morte e suporte emocional a pacientes e familiares^16^. Portanto, corrobora-se a percepgáo dos participantes em relagáo ao desafio que manejo destes pacientes e a gestáo do cuidado nestas unidades representam para a enfermagem.

Os participantes referiram uma rotina assistencial de vigilancia constante sobre os doentes da COVID-19, devido a sua instabilidade clínica. No entanto, ponderaram que o transcorrer do tempo lhes conferiu seguranza e maior sensagáo de controle. Estudo sugeriu que os avanzos no conhecimento clínico sobre a COVID-19, somados aos declínios das primeiras ondas de infecgáo, tém contribuido para o controle das demandas em instituigóes hospitalares^17^.

Destaque deve ser dado a vulnerabilidade social, também descrita como um desafio nestes setores. Evidéncias mostram que as disparidades em saúde interferem na morbimortalidade por COVID-19. Aspectos sociais, de raga e demográficos sáo relevantes do ponto de vista epidemiológico^18^. Regióes periféricas e vulneráveis dos grandes centros urbanos, onde há uma maior concentragáo de pobreza, alta densidade demográfica, precárias condigóes de moradia, caréncia de saneamento básico e, por fim, dificuldade de acesso aos servigos de saúde sáo especialmente vulneráveis^5^.

Ainda assim, os trabalhadores de enfermagem relataram experiéncias importantes nestas unidades, destacando os vínculos estabelecidos com pacientes. Durante o processo de hospitalizagáo por COVID-19, os pacientes vivenciaram o distanciamento físico de suas famílias. As equipes de saúde investiram em estratégias de mitigagáo desse isolamento, incluindo as chamadas por telefone ou vídeo^19^. No entanto, estudo evidenciou que os profissionais de enfermagem da linha de frente vivenciaram também sofrimento emocional nos momentos em que os pacientes falavam com seus familiares pela última vez na chamada por vídeo ou telefone^6^.

O manejo das demandas da comunidade também se evidenciou como um desafio no contexto das Urgéncias e Emergéncias e Unidades de Triagem Respiratória. Sabe-se que a demanda trazida pela COVID-19 fragilizou pontos nefrálgicos históricos do servigo público^20^, especialmente nos locais compreendidos como “portas de entrada” da rede, a exemplo das Urgéncias e Emergéncias. A pressáo que a pandemia causou nestes setores pode ter potencializado dilemas já existentes, tais como os ruídos de comunicagáo entre o servigo e a comunidade.

A fragilidade da rede também pode ser considerada ao se reconhecerem os reflexos da desarticulagáo do sistema de saúde nos desfechos clínicos que ocorrem na unidade COVID-19. Sabe-se que a rede busca reduzir a magnitude da pandemia e, consequentemente, diminuir a pressáo sobre as unidades. No entanto, encontram muitos desafíos^21^. Com isso, os desafios dos trabalhadores de enfermagem nestas unidades envolvem elementos que extrapolam os microespagos, pois dizem respeito também ao modo como a rede se estabelece.

A segunda categoria analítica mostra os desdobramentos negativos deste cotidiano no bem-estar e vida pessoal dos profissionais. Estudo de revisáo integrativa evidenciou um aumento nas percepgóes de ansiedade entre profissionais de enfermagem de setores COVID-19^9^. Sabe-se que, nos momentos de crise, a cooperagáo do coletivo de trabalho fortalece suas capacidades de enfrentamento^22^. Sendo assim, a dispersáo das equipes que outrora atuavam de forma integrada pode ter representado um catalizador da ansiedade, uma vez que a realidade desses trabalhadores se dá em um novo setor de trabalho, onde os vínculos de convivéncia e amizade estáo dispersos.

As repercussóes do trabalho no bem-estar incluíam também desconfortos relacionados as medidas de precaugáo contra a disseminagáo do vírus, que incluíam abundante paramentagáo e restrigóes de circulagáo. Isso impacta no descanso, em suas necessidades fisiológicas e no consumo de lanche/ alimentos, causando desconforto e tornando o trabalho ainda mais difícil.

Estudo evidenciou que os profissionais de enfermagem da linha de frente percebiam maior risco de contaminado nos horarios de lanche e repouso, pois nesses momentos, os colegas costumavam flexibilizar medidas de precaugáo^6^. Estas medidas sao importantes para o controle da disseminagáo do virus^23^, no entanto, tornam o turno de trabalho mais desgastante nestas unidades.

Como consequéncia, os profissionais vivenciavam cansado e exaustáo físicos, mentais e emocionais. Em alguns momentos, vontade de abandonar a linha de frente. Estudo transversal realizado com trabalhadores de enfermagem atuantes no enfrentamento da COVID-19 evidenciou associagáo entre o aumento do número de horas de trabalho semanais e maiores níveis de estresse^2^. Portanto, confirma-se que a sobrecarga enfrentada diariamente tem repercussóes para a saúde mental e física dos profissionais.

Os elementos estressores do espado interno do trabalho se somavam aos do mundo externo, em que os participantes se sentiam fragilizados também na condigáo de cidadáos comuns. O preconceito emerge quando a populado cometa a identificar erroneamente o trabalhador da saúde como um disseminador de doengas. Na pandemia da COVID-19, isso foi resultado de uma construgáo social coletiva, repleta de receios e incertezas^24^.

Os próprios participantes compartilhavam em parte esses receios, pois temiam serem vetores de transmissáo do Sars-CoV-2, especialmente no seio doméstico. Estudos evidenciaram que os profissionais de enfermagem sofreram emocionalmente com o isolamento social e familiar acentuado por estar na linha de frente, pois temiam contaminar seus entes queridos^6^^,^^8^.

Por fim, os depoentes refletiram sobre o mito do “profissional herói” construído pelo imaginário social, ponderando sobre o quanto essa representado contrastava com suas próprias vulnerabilidades. Estudo brasileiro evidenciou que o discurso midiático que fortaleceu a construgáo do profissional de saúde como um anjo ou herói náo foi suficiente para que os trabalhadores de enfermagem se sentissem visibilizados, reconhecidos e amparados^8^.

Portanto, se reconhece que o cotidiano dos trabalhadores de enfermagem nas unidades COVID-19 foi marcado por um conjunto de desafios. Diante disso, ressalta-se a necessidade de que a gestáo das instituigóes hospitalares implemente dispositivos que garantam: presenta de forga de trabalho de enfermagem em número adequado, conforme a demanda; formagáo e capacitagáo da categoria para a assisténcia aos doentes da COVID-19; gestáo dos recursos humanos e materiais; desenvolvimento de protocolos, fluxos e rotinas que otimizem o processo de trabalho em saúde e enfermagem^16^.

Somado a isso, sáo importantes agóes de promogáo a visibilidade profissional junto a sociedade para desmistificar a imagem romantizada sobre a profissáo e problematizar a importancia da enfermagem no enfrentamento da pandemia e os impactos sofridos por estes trabalhadores. Isso pode ser facilitado com a disseminagáo de informagóes, bem como agóes institucionais e sociais que apoiem a enfermagem e fomentem a luta por visibilidade e melhores condigóes de trabalho.

## Conclusao

Os achados deste estudo permitem concluiu-se que trabalhadores de enfermagem de unidades COVID-19 vivenciam desafios complexos em seu cotidiano, com repercussóes em sua experiéncia laboral e em sua vida.

Esta pesquisa apresentou como limitagáo as condigóes especiáis sobre as quais as entrevistas foram realizadas: com distanciamento, uso de EPI's e durante o turno de trabalho (portanto, sujeitas a pressáo do tempo para o retorno as atividades). Estas circunstancias podem ter dificultado a interlocugáo e, em alguns casos, o tempo de duragáo de algumas entrevistas.

Os resultados contribuem com um registro importante para o momento histórico da profissáo. Se por um lado a enfermagem sempre enfrentou desafios em seu cotidiano de trabalho, por outro lado a COVID-19 desnudou aspectos nevrálgicos da complexidade e precarizagáo de seu trabalho. O reconhecimento destes elementos sob o pano de fundo da COVID-19 poderá ser especialmente importante para a reflexáo sobre o futuro da profissáo no mundo pós pandémico.
